# Drivers of Insect Community Change along the Margins of Mountain Streams in Serra da Estrela Natural Park (Portugal)

**DOI:** 10.3390/insects14030243

**Published:** 2023-02-28

**Authors:** Ana Ceia-Hasse, Mário Boieiro, Albano Soares, Sandra Antunes, Hugo Figueiredo, Carla Rego, Paulo A.V. Borges, José Conde, Artur R.M. Serrano

**Affiliations:** 1 Centre for Ecology, Evolution and Environmental Changes, Azorean Biodiversity Group, CHANGE—Global Change and Sustainability Institute, Faculty of Sciences, University of Lisbon, 1749-016 Lisbon, Portugal; 2 Centre for Ecology, Evolution and Environmental Changes, Azorean Biodiversity Group, CHANGE—Global Change and Sustainability Institute, Faculty of Agricultural Sciences and Environment, University of the Azores, Angra do Heroísmo, 9700-042 Azores, Portugal; 3 Tagis—Centro de Conservação das Borboletas de Portugal, 7480-152 Avis, Portugal; 4 Centro de Interpretação da Serra da Estrela/Município de Seia, 6270-423 Seia, Portugal

**Keywords:** beta diversity, community structure, elevation gradients, Lepidoptera, Odonata, mountains

## Abstract

**Simple Summary:**

Mountain ecosystems are important biodiversity hotspots since they host many unique species and provide valuable services. In this study, we analyze the diversity patterns of butterflies and odonates in a mountainous area of high conservation value—Serra da Estrela Natural Park (Portugal)—and we assess which factors are responsible for insect community change between study sites. The insects were sampled along 150 m transects near the margins of three mountain streams, at three elevation levels (500, 1000, and 1500 m). Butterfly species richness was lowest at high altitudes, while odonate species richness did not differ between elevations. Interestingly, species replacement drove the changes between butterfly assemblages, while changes in odonate communities were mostly due to species richness differences. Climatic factors, namely temperature and precipitation, were the main drivers of community change between sites for the two insect groups. The study of mountain insect biodiversity is key to further our understanding on the community assembly processes and provides valuable information to help predict the impacts of environmental changes on mountain biodiversity.

**Abstract:**

Mountain ecosystems are important biodiversity hotspots and valuable natural laboratories to study community assembly processes. Here, we analyze the diversity patterns of butterflies and odonates in a mountainous area of high conservation value—Serra da Estrela Natural Park (Portugal)—and we assess the drivers of community change for each of the two insect groups. The butterflies and odonates were sampled along 150 m transects near the margins of three mountain streams, at three elevation levels (500, 1000, and 1500 m). We found no significant differences in odonate species richness between elevations, but marginal differences (*p* = 0.058) were found for butterflies due to the lower number of species at high altitudes. Both insect groups showed significant differences in beta diversity (βtotal) between elevations, with species richness differences being the most important component for odonates (βrich = 55.2%), while species replacement drove the changes between butterfly assemblages (βrepl = 60.3%). Climatic factors, particularly those depicting harsher conditions of temperature and precipitation, were the best predictors of total beta diversity (βtotal) and its components (βrich, βrepl) for the two study groups. The study of insect biodiversity patterns in mountain ecosystems and of the role played by different predictors contribute to further our understanding on the community assembly processes and may help to better predict environmental change impacts on mountain biodiversity.

## 1. Introduction

The analysis of biodiversity patterns and processes is a central theme in ecology and biogeography, playing a relevant role on the understanding of the relationships between organisms and the environment [1]. Considering the unprecedented rates of biodiversity loss and projections indicating that such trends will continue [2], understanding how mountain biodiversity is structured and its drivers is essential for better predicting environmental change impacts on biodiversity and designing effective conservation strategies.

Mountains are important ecosystems because they support high levels of biodiversity and endemicity and provide essential services, such as climate regulation, freshwater provision and purification, and nutrient cycling [3,4,5]. In the last two decades, elevational studies have been increasingly adopted as models for gradient studies in ecology since the effects of changing abiotic and biotic factors on biodiversity can be analyzed across a relatively small geographical area [4,6]. Many studies on mountain insect diversity have focused on the assessment of species richness change along elevation for different taxa and spatial scales and on the identification of environmental predictors of alpha diversity [7,8,9,10]. However, there is still limited knowledge on how insect communities change across elevation (beta diversity), not only on the contributions of species richness differences and species turnover, but also on the relative roles of spatial and environmental factors as drivers of community change [11,12,13]. Several studies report that community change along elevational gradients mostly results from species turnover, usually driven by elevational, climatic, and topographic factors [12,13,14,15]. For example, Fontana and colleagues [16] showed that turnover drives the differences in beta diversity of multiple taxa (including ants, beetles, butterflies, and grasshoppers) in the European Alps, with community changes increasing with elevational distance, but without marked community boundaries along the gradient. Additionally, various studies carried at the Espinhaço Mountain Range (Brazil) identified turnover as the most important component of beta diversity for different insect taxa (ants, butterflies, dung beetles, termites, and wasps) with elevation, climate, and vegetation variables being important predictors of community change [14,15,17,18,19].

Insects are highly diverse and vital for ecosystem functioning, playing key ecological roles as pollinators, herbivores, decomposers, predators, and prey [20,21]. Recent studies suggest that insect diversity, abundance, and biomass are alarmingly declining due to several threats, particularly habitat loss and degradation, climate change, invasive species, and pollution [22,23,24,25,26]. Mountain insect diversity is quite vulnerable to these threats due to the narrow range of many species, their specialized ecological interactions, and their specific ecophysiology (e.g., cold-adapted species) [27,28]. Consequently, in the last few years, reports have been accumulating on projected mountain population/species extinctions, species range contractions, and elevational shifts [29,30,31,32,33]. The situation is worrisome for many insect taxa in different mountain ranges as shown by a study at Sierra de Guadarrama (Spain), where butterfly species richness has declined significantly in the last 30 years (particularly at low elevations), the communities shifted nearly 300 m uphill as a response to climate warming, and considerable species losses are expected in the coming years [29]. For these reasons, many authors stress that it is urgent to increase our knowledge on insect mountain biodiversity, implement biodiversity monitoring programs, and set local-specific and scientific-based conservation strategies to mitigate or halt biodiversity losses of the most vulnerable taxa.

Butterflies and odonates are considered good bioindicators for biodiversity monitoring since these organisms are easy to sample and identify, their biology is well-known, and their responses to environmental change are predictable and representative of the community [34,35]. Therefore, butterflies have often been selected as model organisms in mountain biodiversity studies [4,13,15,16,31,36]. Butterflies are insect herbivores, with the caterpillars feeding on the plant tissues of a few to many host species, while the adults usually feed on nectar. Several studies highlight the role of these organisms as efficient and effective bioindicators of habitat and climate change, often outperforming other animal groups [4,37]. Odonates have a very different life history from butterflies (they are generalist predators with aquatic larval stages and terrestrial adults) and have been effectively used to monitor freshwater systems (i.e., both in water and along riparian corridors) [32,34,38,39,40]. The combined use of these two insect indicator groups in our study can provide more robust results and may allow for testing the generality of community assembly rules and of biodiversity responses to environmental change [41,42,43].

The objectives of this study were to assess and compare alpha and beta taxonomic diversity metrics of butterflies and odonates across elevations in Serra da Estrela. More specifically, we aimed to evaluate the roles of species replacement and species richness differences in generating beta diversity patterns and to assess the relative importance of spatial and environmental factors as drivers of insect community change.

## 2. Materials and Methods

### 2.1. Study Area

This study took place in Serra da Estrela (N 40º 19′ 18.47″, W 7º 36′ 49.81″), the highest mountain in continental Portugal (with 1993m). Serra da Estrela includes the western extreme of the Iberian Central System, which is considered one of the main mountain systems in the Iberian Peninsula. Serra da Estrela has been classified as Natural Park since 1976, is part of the Natura 2000 network, and its upper areas are included in the Ramsar Convention [44]. The study area is characterized by Atlantic and Mediterranean climates and different biogeographic regions, being a particularly important area for several species and habitats associated with high elevation in Portugal [44]. Among the butterfly and odonate species occurring in this region, there are several Iberian endemic, rare, or endangered species [45,46,47], and some are protected by law at the international level (Habitats Directive 92/43/EEC, Council of the European Communities) [48].

### 2.2. Data Collection: Butterfly and Odonate Sampling

**Figure 1:**
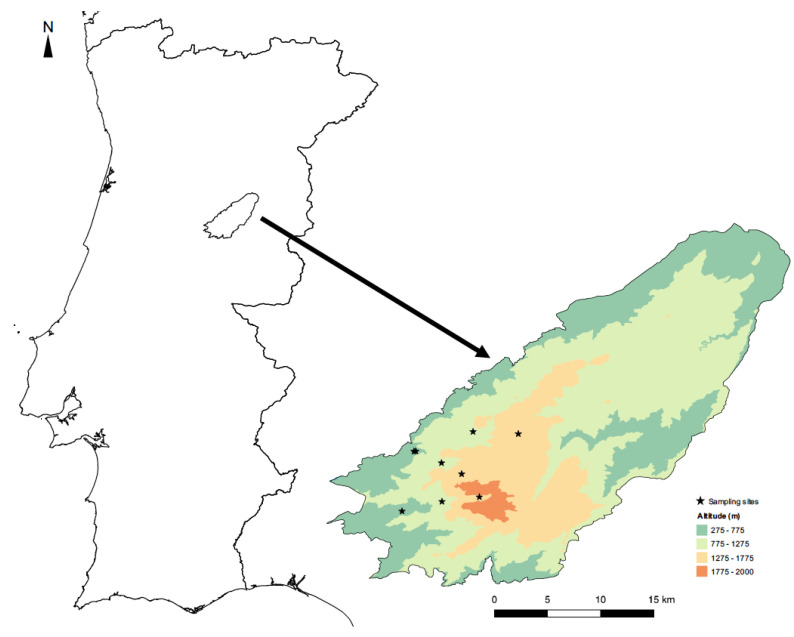
Location of the Serra da Estrela Natural Park in Portugal, showing the distribution of the sampling sites by elevational band in this protected area.

Sampling took place at three elevations (500, 1000, and 1500 m) along the margins of three mountain streams belonging to the same water basin—the Mondego river (Figure 1; Table S1)—thus allowing us to evaluate the influence of elevation on insect communities. Adult dragonfly and damselfly (Odonata) and diurnal butterfly (Lepidoptera, Rhopalocera) species were identified visually using the fixed transects method, which has been frequently applied in biodiversity studies since it gives good estimates of species richness and abundance at the local scale [36,49]. The study lasted two consecutive years (2013 and 2014) with the transects being conducted monthly along the mountain stream margins (up to 50 m from the watercourse), from June to September, since this is the activity period for most species of the two study groups.

Each transect had a length of 150 m, and the study insects were recorded up to 2.5 m to each side and 5 m ahead of the recorder. Sampling was carried out between 10 a.m. and 6 p.m. under favorable climatic conditions. During hot weather periods and rainy, windy, and cloudy days the sampling did not take place. Individuals were identified to species on the spot, but occasionally some individuals were captured to confirm species identity, being immediately released afterwards. The necessary permits for insect sampling in Serra da Estrela Natural Park were obtained from the Instituto para a Conservação da Natureza e das Florestas (ICNF, Portugal).

### 2.3. Data Analysis

We pooled the data for each study site and calculated alpha diversity metrics separately for butterflies and odonates following the Hill numbers [50,51]. Hill numbers combine information on species richness, rarity, and dominance, and they differ in their calculation only by an exponent q that determines their sensitivity to species relative abundances. We computed the observed species richness (q = 0), the exponential of Shannon–Wiener diversity index (q = 1), the reciprocal of Simpson’s diversity index (q = 2), and the reciprocal of the Berger–Parker index (i.e., the reciprocal of the proportional abundance of the most common species) (q = ∞) [51]. We further presented the overall number of individuals collected at each site and calculated the Smith and Wilson evenness index. We estimated species richness with the non-parametric estimator Chao1 and assessed sampling completeness per site as the ratio of observed to estimated species richness. Differences in alpha diversity metric values between elevations and mountain streams were analyzed separately for each insect group using non-parametric Kruskal–Wallis tests followed by Dunn multiple comparison post hoc tests.

The differences in species composition between communities were assessed by beta diversity analysis using Jaccard’s index and following the partition of total beta diversity (βtotal) into its two components: βrepl (the component assessing variation due to species replacement) and βrich (the component assessing variation due to species richness differences), where βtotal = βrepl + βrich [52,53]. We tested for statistically significant differences in each component of beta diversity between elevations and mountain streams, for each insect group separately using the analysis of similarities (ANOSIM). Then, to evaluate the influence of environmental and spatial factors on beta diversity patterns, we performed variation partition based on redundancy analysis [54]. For the environmental factors, we considered site elevation and bioclimatic variables related to temperature and precipitation retrieved from the WorldClim database [55] (Table S2), while for the spatial factors, we considered sampling site coordinates. We used distance-based Moran’s eigenvector maps (dbMEM) to represent spatial relationships among sites [56]. In this analysis, a matrix of geographic distances among sampling sites is constructed, and then the spatial explanatory variables that are used in the variation partition analysis are the eigenvectors obtained from a principal coordinate analysis performed upon the matrix of geographic distances. The eigenvectors represent wide- and small-scale variation: the first dbMEM vectors represent large-scale variation, while later dbMEM vectors describe small-scale variation. To select the variables that explain beta diversity patterns (from the environmental and spatial variables initially considered), we used forward selection [57] to create more parsimonious sets of explanatory variables that were then included in the variation partition analysis. The percentage of variation in beta diversity explained by pure and shared effects was estimated using adjusted R2 values [58], and its significance assessed with permutation tests [54]. When an effect had a negative adjusted R2, such a value was interpreted and presented as zero. This is because such negative values indicate that less variation is explained than by random explanatory variables. In these cases, the sum of pure and shared effects does not equal the total variation explained, because total variation includes the negative value [59]. Statistical analyses were performed using packages *BAT* [60], *dunn.test* [61], *vegan* [62], *adespatial* [63], and *stats* within an R environment [64].

## 3. Results

### 3.1. Alpha Diversity Patterns

**Figure 2:**
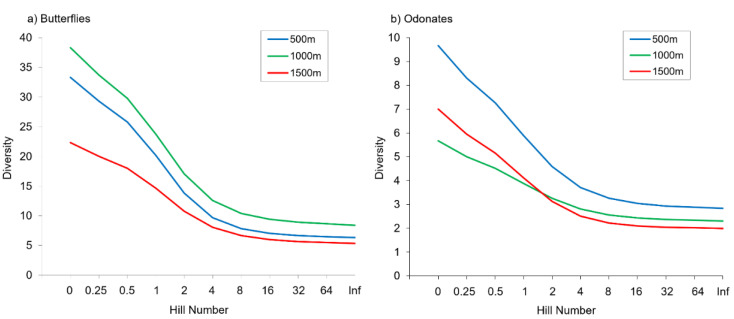
Alpha diversity of butterflies (**a**) and odonates (**b**) at the different elevations represented by Hill numbers.

We recorded a total of 1680 individuals from 66 species of butterflies, and 796 individuals of 24 odonate species in the nine study sites (Tables S3 and S4). Two dragonflies (*Macromia splendens* and *Oxygastra curtisii*) and one butterfly (*Euphydryas aurinia*) are protected in Portugal, and several other species (like *Aeshna juncea*, *Sympetrum sanguineum*, *Apatura ilia*, *Cyaniris semiargus*) are considered rare being restricted to Serra da Estrela or to a few other areas in this country [65]. Sampling completeness was high in all study sites ranging from 0.73 to 1.00 for butterflies and 0.88 to 1.00 for odonates (Tables S5 and S6). Alpha diversity per site was always higher for butterflies than for odonates as expected, since the former is a species-rich insect group in many terrestrial ecosystems (Table 1, Tables S5 and S6). Odonate species richness did not differ between elevations (*p* = 0.223), but marginally significant differences in butterfly species richness were detected along the elevational gradient (*p* = 0.058). No statistically significant differences between elevations were found in any of the other alpha diversity metrics, except for butterfly abundance and evenness (*p* < 0.05) (Figure 2). Butterflies were more abundant at intermediate than at higher elevations. We also found no significant differences in alpha diversity metrics between streams for the two insect groups (Figure S1), but evenness changed significantly between sites for both butterflies and odonates.

### 3.2. Beta Diversity Patterns and Drivers

In the study area, the total beta diversity (βtotal) for butterflies was 0.58 ± 0.12 (mean ± SD) with different contributions from the components due to species replacement (βrepl = 0.35 ± 0.15) and due to species richness differences (βrich = 0.23 ± 0.17). In fact, the species replacement component showed on average a larger contribution (βrepl = 60.3%) to the βtotal of butterflies. For odonates, the total beta diversity (βtotal) was 0.59 ± 0.21 (mean ± SD), and the contributions due to species replacement (βrepl = 0.26 ± 0.25) and due to species richness differences (βrich = 0.33 ± 0.20) were more similar. On average, βrich contributed to 55.2% of βtotal for odonates. Interestingly, we found that βtotal differed between elevations for both butterflies and odonates (*p* = 0.016 and *p* = 0.006, respectively), but the changes in the two insect assemblages were driven by different processes. Butterfly βrich differed significantly between elevations (*p* = 0.043) and βrepl did not (*p* = 0.364), while for odonates, significant differences were found between elevations in βrepl (*p* = 0.048) but not in βrich (*p* = 0.173). Furthermore, for both butterflies and odonates, we found no significant differences on βtotal nor on its components (βrepl, βrich) between sites at the same elevational level (all *p* > 0.45).

**Figure 3:**
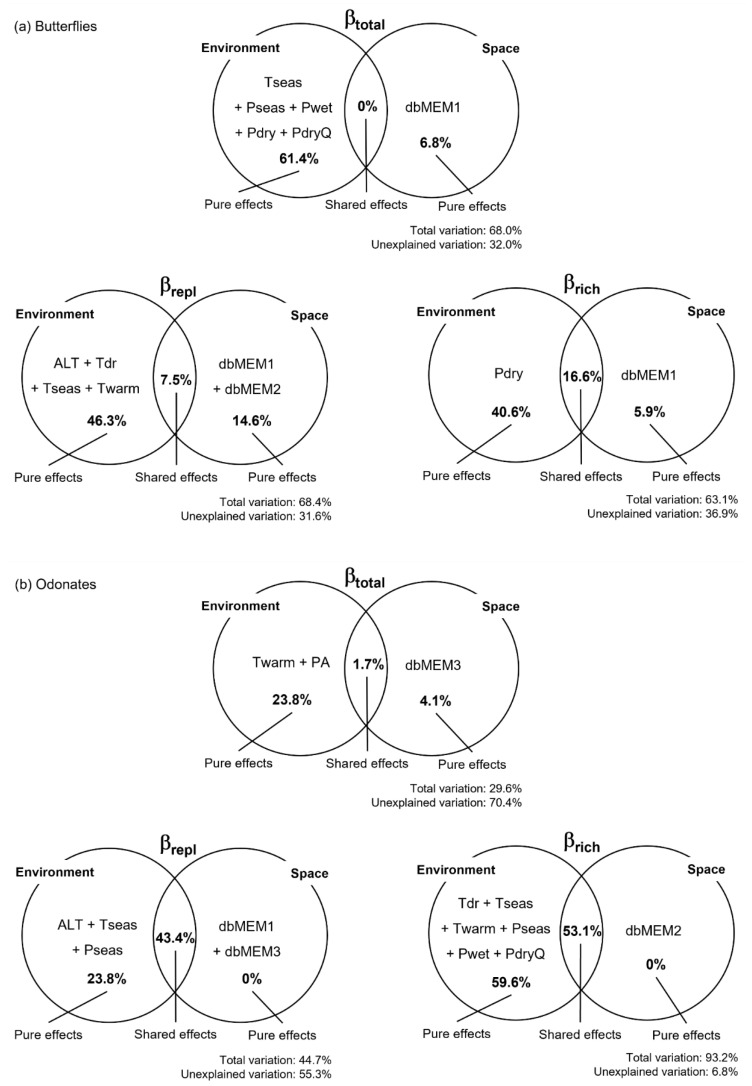
Variation partition for butterflies (**a**) and odonates (**b**). Venn diagrams show the variables explaining variation in βtotal, βrepl, and βrich, as well as the percentage of variation (adjusted R2) explained by each effect. Variable names stand for: altitude (ALT), annual precipitation (PA), precipitation of the driest month (Pdry), precipitation of the driest quarter (PdryQ), precipitation seasonality (Pseas), precipitation of the wettest month (Pwet), temperature mean diurnal range (Tdr), temperature seasonality (Tseas), and maximum temperature of the warmest month (Twarm). dbMEM variables correspond to the spatial relationships among sampling sites.

To identify the drivers of butterfly and odonate beta diversity in the study area, we assessed the influence of environmental and spatial factors and their combined effects. From the environmental factors, several climate variables related with temperature and precipitation were selected as explanatory (Table S2). The total variation in beta diversity and its components explained by the selected variables was relatively high and homogeneous for butterflies and more heterogeneous for odonates (Figure 3). Climate variables play a major role as predictors of variation in the species richness component (βrich) and in total beta diversity (βtotal) between sites and, jointly with elevation, explain a considerable fraction of variation in the turnover component (βrepl) for the two insect groups. Overall, pure spatial factors seem to be less important as drivers of variation in odonate beta diversity and its components than for butterflies, and their influence was noticed at different spatial scales for the two insect groups (e.g., at finer spatial scales for odonates). Nevertheless, the two components of odonate beta diversity were strongly influenced by spatial-structured environmental effects, which accounted for a large fraction of the explained variation (Figure 3).

## 4. Discussion

### 4.1. Alpha Diversity Patterns

A high number of studies show that species richness is lower at higher elevations, with the most common patterns for different plant and animal groups corresponding to a monotonic decrease of species richness with elevation, or to a mid-elevation peak pattern, where species richness is higher at intermediate elevations [66,67]. In contrast with these patterns, in our study, we did not observe significant differences in odonate species richness between elevations, which was in part due to the heterogeneity of the results found at low and high elevations, where both species-poor and species-rich communities were found. Several rare species, including the protected *Macromia splendens* and *Oxygastra curtisii*, were only found in one species-rich site at low elevation, while six other drangonfly species, including three *Sympetrum* species, were exclusive to a single high elevation site. Local and landscape-scale variables (e.g., climate, vegetation structure, human disturbance) play an important role in determining local odonate assemblages [68,69,70] and may have contributed to the heterogeneity of the results found within elevational levels.

Butterfly species richness followed a mid-elevation peak pattern and showed marginally significant differences between elevations. The pattern was common to the three study gradients and resulted from a much lower number of species at higher elevations, including the absence of some species that were frequent and abundant at mid-elevation, like *Maniola jurtina*, *Melitaea deione*, *Pieris brassicae*, *P. napi,* and *Satyrium spini*. Butterfly species richness and abundance have been reported to be lowest at high elevations in most studies due to geometric constrains (smaller area), more severe environmental conditions (i.e., harsh climatic conditions *sensu* [71]), and lower productivity and resource availability for larval development [1,72,73]. In addition to temperature and precipitation, solar radiation, oxygen availability, and wind turbulence also influence insect occurrence at higher elevations [74]. Decreasing patterns in species richness with elevation are also commonly found in other arthropod groups, such as ants [75] and beetles [76]. On Serra da Estrela, the area above 1500 m is reduced (nearly 80km^2^) when compared with lower altitudinal bands, ice cover duration has wide annual fluctuations but may last up to six months, and average minimum temperatures fall below zero during several months [77]. Additionally, the dominant vegetation types (altitudinal grasslands and subalpine heathland with dwarf junipers) have lower plant species diversity and a more simplified structure. These conditions pose serious obstacles to the survival of many butterfly species, but some (36 out of the 66 recorded) still find suitable conditions to occur. The reasons for the absence of a marked decrease in species richness along elevation for both study groups may be due to the short elevational range studied and the relatively low upper limit of the study gradient (1500 m) that does not pose challenging conditions for the occurrence of many butterfly and odonate species. It is also important to stress that most butterflies and odonates (e.g., dragonflies) have good dispersal ability when compared with other insect groups and may cover larger elevational ranges [78]. Their movements may even be eased, if they move across areas of similar habitat as usually happens along the margins of mid-elevation mountain streams. Nevertheless, these results merit further investigation by considering a higher number of study gradients and additional data points across the full extent of elevations in the study area, and by specifically testing the ecological hypotheses proposed to explain species richness variation across elevations, such as the elevational Rapoport’s rule [79] or the mid-domain effect hypothesis [80].

### 4.2. Beta Diversity Patterns

While in general we found no significant differences between elevations in alpha diversity metrics for both butterflies and odonates, analyzing beta diversity patterns provided insights into the assembly mechanisms of the two insect groups. Interestingly, we found that beta diversity was driven by different processes in butterflies and odonates: in the former, species replacement between sites (βrepl) was the most important component of beta diversity, while for the latter, the differences in species richness (βrich) were the most important.

Species replacement is usually the most important process driving beta diversity of many insect groups in mountain ecosystems, including for butterflies [12,13,14,15,16,81,82]. The changes in abiotic and biotic conditions along the elevation gradient may act as habitat filters determining differences in species composition between mountain sites. Many studies have emphasized the role of altitude and climatic variables as drivers of species turnover in mountains, but other abiotic and biotic variables (often correlated with the previous) may also be important mechanisms of community assembly [74,83].

We found that pure climate variables related to temperature and precipitation, particularly those informing on the more extreme conditions, seem to play the major role on driving butterfly and odonate beta diversity. Butterfly beta diversity was mostly explained by differences between sites in the warmest, wettest, and driest periods, suggesting that specific values are tolerated by some species, but not by others. Temperatures of the warmest periods of the year were also responsible for the changes in the odonate assemblages (Figure 3). Pure environmental effects were the major drivers of butterfly and odonate beta diversity in Serra da Estrela, while spatial-structured environmental variation strongly influenced the two components of odonate beta diversity (but not βtotal). We found some heterogeneity of climatic conditions within elevation bands (driven by topographical, geomorphological, and biophysical processes) that influenced the insect assemblages, particularly odonates. Climatic variables (e.g., temperature) affect insect assemblages directly by influencing species survival, foraging, and reproductive performance, as well as indirectly via effects on food resource availability and vegetation composition and structure [84]. For example, in our study, the thermophilic *Charaxes jasius* was restricted to the lower altitudinal band, where its host plant (*Arbutus unedo*) occurs, and warmer conditions allow for its activity.

Nevertheless, several studies have stressed that environmental factors other than climate variables may act at different spatial scales driving changes in species richness and composition of arthropod assemblages [13,15,36,85]. Plant diversity, composition, and structure may influence the distribution and abundance of butterflies and other herbivore insects since plants provide food resources and habitat for both larvae and adults [15,86,87]. Additionally, vegetation structure and prey availability may drive local adult odonate diversity, while physical, chemical, and biological characteristics of streams are known to influence larvae species richness and composition [41,42]. Thus, the comprehensive assessment of insect biodiversity should ideally include a large set of environmental variables that potentially influence the different life stages of the study groups. Since the effects of these environmental variables are often confounded with elevation, it will be critical to assess them with a proper sampling design [87]. In general, pure spatial factors played a minor role as drivers of beta diversity (βtotal and its components) for the two insect groups but seemed to have a somewhat higher influence on butterfly than in odonate assemblages.

Our study suggests that environmental filtering, particularly climate variables indicative of harsher conditions, is the main process shaping the changes in species richness and composition of butterfly and odonate assemblages in Serra da Estrela. These findings further our understanding of the mechanisms that govern species distribution along elevation gradients and may help predict the consequences of global warming on mountain biodiversity. Mountain ecosystems are biodiversity hotpots, harboring unique endemic species, and a disproportionate high number of species considering their area [88]. During the last few decades, evidence has accumulated on the negative consequences of climate warming on mountain biodiversity with several species facing range contractions and local population extinctions [29,89,90]. The occurrence of several range-restricted insect species at mid- and high elevations on Serra da Estrela emphasizes the need to implement a monitoring program to track changes on species abundances and distributions.

## 5. Conclusions

The Serra da Estrela Natural Park is one of the most species-rich protected areas in Portugal, including several rare butterflies and odonates. Butterfly species richness was lowest at high elevations due to the harsher environmental conditions that affect their survival, foraging, and reproduction, but also limit the occurrence of host plants. Odonates are generalist predators, and their species richness showed no association with elevation but seemed to be influenced by local habitat characteristics. We found that changes in butterfly assemblages were mainly due to compositional differences between sites, while for odonates, they were due to differences in species richness. Local climatic conditions, particularly temperature and precipitation, are the main drivers of variation in both butterfly and odonate assemblages in Serra da Estrela. However, further studies including other environmental factors as predictors of beta diversity, such as land-use information, vegetation structure, diversity and abundance of butterfly host plants, and prey availability for odonates, are necessary to increase our understanding on the drivers of Serra da Estrela biodiversity [36,43,91,92]. Additionally, it will be crucial to assess the changes in butterfly and odonate functional diversity along elevation since species traits may provide complementary information to species richness on community assembly processes [93]. Finally, our findings highlight the need to implement a long-term monitoring plan to assess the effects of predicted climate changes on Serra da Estrela biodiversity, aiming to support decision making on the conservation management of rare and threatened mountain insect species.

## Figures and Tables

**Table 1:** Alpha diversity metrics (range and mean ± SD) for the two insect groups across study sites. Species Richness: observed species richness, EShannon: exponential of Shannon diversity index, ISimpson: reciprocal of Simpson’s diversity index, Berger–Parker: reciprocal of Berger–Parker index, Estimated Species Richness: estimated species richness using Chao1.

	Butterflies	Odonates
Species Richness	16–48 (31.3 ± 8.9)	3–14 (7.4 ± 3.2)
Abundance	88–409 (186.7 ± 96.0)	17–164 (88.4 ± 48.3)
EShannon	10.3–33.0 (19.5 ± 6.1)	2.1–8.4 (4.6 ± 1.7)
ISimpson	7.2–26.2 (13.9 ± 15.2)	1.8–6.1 (3.7 ± 1.3)
Berger–Parker	0.07–0.27 (0.17 ± 0.06)	0.30–0.71 (0.45 ± 0.13)
Evenness	0.48–0.61 (0.53 ± 0.04)	0.27–0.74 (0.43 ± 0.16)
Estimated Species Richness	16.0–53.6 (36.2 ± 10.9)	3.0–15.5 (7.8 ± 3.6)
Completeness	0.73–1.00 (0.88 ± 0.08)	0.88–1.00 (0.97 ± 0.05)

## Data Availability

All relevant data on butterfly and odonate diversity are within the paper or the Supplementary Materials and available from GBIF (http://ipt.gbif.pt/ipt/resource?r=odonata_estrela_portugal and http://ipt.gbif.pt/ipt/resource?r=lepidoptera_estrela, accessed on 9 January 2022). Climate data are available from the WorldClim database (http://worldclim.org/version2, accessed on 3 October 2022).

## Supplementary Materials

The following supporting information can be downloaded at: https://www.mdpi.com/article/10.3390/insects14030243/s1, Figure S1: Alpha diversity per stream for butterflies (**a**) and odonates (**b**) represented by Hill numbers; Table S1: Geographic information on sampling sites from Serra da Estrela Natural Park; Table S2: Bioclimatic variables tested and selected as explanatory in the variation partition analysis of butterfly and odonate beta diversity, namely total beta diversity and its components assessing species replacement and species richness differences; Table S3: Butterfly species occurrence at study sites on the margins of different mountain streams and elevation levels; Table S4: Odonate species occurrence at study sites on the margins of different mountain streams and elevation levels; Table S5: Butterfly alpha diversity in the study sites on the margins of mountain streams at different elevation levels; Table S6: Odonate alpha diversity in the study sites on the margins of mountain streams at different altitudinal levels.
