# Supplementary material for: Drivers of Insect Community Change along the Margins of Mountain Streams in Serra da Estrela Natural Park (Portugal)
Source: Insects. 2023 Feb 28;14(3):243. doi: 10.3390/insects14030243 (PMC10058670; doi:10.3390/insects14030243)
Supplement: Supplementary file 1 [file insects-14-00243-s001.zip › insects-2200100-supplementary.pdf]

Supporting Information

Table S1. Geographic information on sampling sites (site name, mountain stream, elevation and geographical coordinates) from Serra da Estrela Natural Park.

Site name	Mountain stream	Elevation (m)	Latitude (N)	Longitude (W)
Fervença	Fervença	1411	40.404	−7.591
Sabugueiro	Fervença	1028	40.406	−7.641
Jugais Alva	Fervença	622	40.385	−7.706
Pragueira	Caniça	1584	40.360	−7.653
Porto do Boi	Caniça	1010	40.372	−7.676
Jugais Caniça	Caniça	614	40.385	−7.704
Nave	Loriga	1637	40.334	−7.634
Loriga	Loriga	926	40.329	−7.675
Cabeça	Loriga	512	40.319	−7.719

Table S2. Bioclimatic variables (and their acronyms) tested and selected as explanatory in the variation partition analysis of butterfly and odonate beta diversity, namely total beta diversity (β_{total}) and its components assessing species replacement (β_{repl}) and species richness differences (β_{rich}).

Variable (acronym)	Butterflies	Odonates
Annual Mean Temperature (TA)	-	-
Mean Diurnal Range (Tdr)	β_{repl}	β_{rich}
Temperature Seasonality (Tseas)	β_{total}, β_{repl}	β_{repl}, β_{rich}
Maximum Temperature of the Warmest Month (Twarm)	β_{repl}	β_{total}, β_{rich}
Minimum Temperature of the Coldest Month (Tcold)	-	-
Mean Temperature of the Warmest Quarter (TwarmQ)	-	-
Mean Temperature of the Coldest Quarter (TcoldQ)	-	-
Annual Precipitation (PA)	-	β_{total}
Precipitation Seasonality (Pseas)	β_{total}	β_{repl}, β_{rich}
Precipitation of the Wettest Month (Pwet)	β_{total}	β_{rich}
Precipitation of the Driest Month (Pdry)	β_{total}, β_{rich}	-
Precipitation of the Wettest Quarter (PwetQ)	-	-
Precipitation of the Driest Quarter (PdryQ)	β_{total}	β_{rich}

Table S3. Butterfly species occurrence (1=present; 0=not observed) at study sites on the margins of different mountain streams (A- Fervença, B - Caniça and C- Loriga) and elevation levels (500m, 1000m and 1500m). Butterfly species are listed alphabetically.

Elevation (m)	500	500	500	1000	1000	1000	1500	1500	1500
**Mountain stream**	**A**	**B**	**C**	**A**	**B**	**C**	**A**	**B**	**C**
Butterfly species									
Aglais io	0	1	0	0	0	0	0	0	0
Aglais urticae	0	0	1	1	0	0	0	1	0
Apatura ilia	0	1	0	0	0	0	0	0	0
Argynnis pandora	1	1	1	1	1	1	1	1	1

Argynnis paphia	1	1	0	0	1	0	0	0	0
Aricia cramera	0	0	1	1	1	1	1	1	0
Brintesia circe	1	1	0	1	1	1	1	1	0
Celastrina argiolus	1	1	0	1	1	1	1	1	1
Charaxes jasius	0	0	1	0	0	0	0	0	0
Coenonympha dorus	0	0	0	0	1	0	0	0	0
Coenonympha pamphilus	1	1	0	0	0	0	1	0	0
Colias crocea	1	1	1	1	1	1	1	1	1
Cyaniris semiargus	0	0	0	0	1	1	0	1	0
Euphydryas aurinia	0	0	0	0	0	1	0	0	0
Fabriciana adippe	1	1	0	1	1	0	1	0	1
Glaucopsyche melanops	0	0	0	1	0	0	0	0	0
Gonepteryx rhamni	1	1	1	1	1	1	1	0	1
Hesperia comma	1	1	0	1	1	0	1	1	0
Hipparchia fidia	0	1	0	0	1	1	0	0	0
Hipparchia hermione	1	1	0	1	1	1	1	1	0
Hipparchia semele	1	1	1	1	1	1	0	0	0
Hipparchia statilinus	0	0	0	0	1	1	1	0	0
Hyponephele lycaon	0	1	0	0	1	0	1	1	0
Iphiclides feisthamelii	0	0	1	0	1	1	0	0	0
Issoria lathonia	0	0	0	0	1	0	1	1	1
Laeosopis roboris	1	1	1	0	0	0	0	0	0
Lampides boeticus	0	0	0	0	1	0	1	0	0
Lasiommata megera	1	1	1	1	1	1	0	1	0
Leptidea sinapis	1	1	1	1	1	1	1	0	0
Leptotes pirithous	1	1	1	1	1	1	1	1	0
Limenitis reducta	1	1	1	1	0	0	0	0	0

Table S3. (continued). Butterfly species occurrence (1=present; 0=not observed) at study sites on the margins of different mountain streams (A- Fervença, B - Caniça and C- Loriga) and elevation levels (500m, 1000m and 1500m). Butterfly species are listed alphabetically.

**Elevation (m)**	**500**	**500**	**500**	**1000**	**1000**	**1000**	**1500**	**1500**	**1500**
**Mountain stream**	**A**	**B**	**C**	**A**	**B**	**C**	**A**	**B**	**C**
Butterfly species									
Lycaena alciphron	1	0	1	1	1	0	0	0	1
Lycaena bleusei	0	0	1	0	1	0	0	0	0
Lycaena phlaeas	1	1	1	1	1	1	1	0	1
Lycaena tityrus	0	0	1	0	1	0	0	0	0
Maniola jurtina	0	0	1	1	1	1	0	0	0
Melanargia lachesis	1	1	1	1	1	1	1	1	1
Melanargia occitanica	0	0	0	0	1	0	0	0	0
Melanargia russiae	0	0	0	0	1	0	0	0	0

Melitaea deione	1	1	1	1	1	1	0	0	0
Melitaea nevadensis	1	0	0	1	1	1	1	1	0
Melitaea parthenoides	0	0	1	0	0	1	0	0	0
Melitaea phoebe	0	0	0	0	1	1	0	0	0
Melitaea trivia	0	0	0	0	1	1	0	0	0
Nymphalis antiopa	0	1	1	1	0	0	0	0	1
Ochlodes sylvanus	1	1	0	0	1	0	0	0	0
Pararge aegeria	1	1	1	1	1	1	1	0	1
Pieris brassicae	1	1	1	1	1	1	0	0	0
Pieris napi	1	1	1	1	1	1	0	0	0
Pieris rapae	1	1	1	1	1	1	1	1	1
Plebejus argus	0	0	0	1	1	0	1	1	1
Polygonia c-album	1	1	0	0	0	0	0	0	0
Polyommatus icarus	1	1	1	1	1	0	1	0	0
Pontia daplidice	0	1	1	1	1	1	0	1	1
Pyrgus malvoides	0	0	0	1	0	0	0	1	0
Pyronia cecilia	0	0	1	0	1	0	0	0	0
Pyronia tithonus	1	1	1	1	1	1	1	1	0
Satyrium esculi	0	0	1	0	0	0	0	0	0
Satyrium spini	1	1	1	1	1	0	0	0	0
Satyrus actaea	1	1	0	0	0	0	0	1	1
Speyeria aglaja	0	0	0	0	1	0	1	0	0
Thymelicus acteon	0	0	1	0	1	0	0	0	0
Thymelicus lineola	0	0	0	0	0	1	1	0	0
Thymelicus sylvestris	1	0	0	1	1	1	1	0	1
Vanessa atalanta	0	0	0	1	0	0	1	0	0
Vanessa cardui	0	0	0	0	0	0	1	1	0

Table S4. Odonate species occurrence (1=present; 0=not observed) at study sites on the margins of different mountain streams (A- Fervença, B - Caniça and C- Loriga) and elevation levels (500m, 1000m and 1500m). Odonate species are listed alphabetically.

Elevation (m)	500	500	500	1000	1000	1000	1500	1500	1500
**Mountain stream**	**A**	**B**	**C**	**A**	**B**	**C**	**A**	**B**	**C**
Odonate species									
Aeshna cyanea	1	1	1	0	0	0	0	1	1
Aeshna juncea	0	0	0	0	1	0	0	0	1
Anax imperator	0	0	0	0	0	0	0	1	0
Boyeria irene	1	1	1	1	1	1	0	0	0
Calopteryx haemorrhoidalis	0	0	1	0	0	0	0	0	0
Calopteryx virgo	1	1	1	1	1	1	1	0	1
Calopteryx xanthostoma	0	0	1	0	0	0	0	0	0
Cordulegaster boltonii	1	1	1	1	1	1	1	0	1
Ischnura graellsii	0	0	0	0	0	0	0	1	0
Lestes dryas	0	0	0	0	0	0	0	1	1
Lestes virens	0	0	0	0	0	0	0	1	0

Lestes viridis	0	1	1	0	0	0	0	0	0
Libellula depressa	0	0	1	0	0	0	0	0	0
Libellula quadrimaculata	0	0	0	0	0	0	0	1	1
Macromia splendens	0	0	1	0	0	0	0	0	0
Onychogomphus uncatus	1	0	1	1	1	1	0	0	1
Orthetrum coerulescens	1	0	1	0	0	0	0	0	0
Oxygastra curtisii	0	0	1	0	0	0	0	0	0
Platycnemis latipes	0	1	1	0	0	1	0	0	0
Pyrrosoma nymphula	1	1	1	1	1	1	1	1	1
Sympetrum flaveolum	0	0	0	0	0	0	0	1	0
Sympetrum fonscolombii	0	0	0	0	0	0	0	1	0
Sympetrum sanguineum	0	0	0	0	0	0	0	1	0
Sympetrum striolatum	0	1	0	0	0	0	0	0	0

Table S5. Butterfly alpha diversity in the study sites on the margins of mountain streams (A- Fervença, B - Caniça and C- Loriga) at different elevation levels (500m, 1000m and 1500m). SR: observed species richness, Abund: abundance, EShan: exponential of Shannon diversity index, ISimp: reciprocal of Simpson's diversity index, BP: reciprocal of Berger-Parker index, SR_{estim}: estimated species richness, Comp: completeness.

Elevation (m)	Stream	SR	Abund	EShan	ISimp	BP	Evenness	SR_{estim}	Comp
500	A	32	188	20.0	13.6	0.16	0.54	39	0.82
500	B	35	167	21.3	14.6	0.16	0.53	48.2	0.73
500	C	33	175	19.3	13.3	0.15	0.50	36.6	0.90
1000	A	35	220	20.6	14.7	0.13	0.48	38.5	0.91
1000	B	48	409	33.0	26.2	0.07	0.48	53.6	0.90
1000	C	32	211	17.7	10.4	0.25	0.51	34.1	0.94
1500	A	29	132	18.7	13.2	0.18	0.56	32.5	0.89
1500	B	22	88	15.0	12.0	0.15	0.56	27.6	0.80
1500	C	16	90	10.3	7.2	0.27	0.61	16	1

Table S6. Odonate alpha diversity in the study sites on the margins of mountain streams (A- Fervença, B - Caniça and C- Loriga) at different altitudinal levels (500m, 1000m and 1500m). SR: observed species richness, Abund: abundance, EShan: exponential of Shannon diversity index, ISimp: reciprocal of Simpson's diversity index, BP: reciprocal of Berger-Parker index, SR_{estim}: estimated species richness, Comp: completeness.

Elevation (m)	Stream	SR	Abund	EShan	ISimp	BP	Evenness	SR_{estim}	Comp
500	A	7	70	5.1	4.5	0.3	0.34	8	0.88
500	B	8	105	4.2	3.1	0.5	0.33	8	1
500	C	14	148	8.4	6.1	0.32	0.43	15.5	0.90
1000	A	5	82	4.1	3.6	0.4	0.74	5	1
1000	B	6	101	3.7	3.1	0.42	0.3	6	1
1000	C	6	79	3.9	3.1	0.5	0.36	6	1
1500	A	3	17	2.1	1.8	0.71	0.49	3	1
1500	B	10	164	4.3	2.9	0.54	0.27	10.3	0.98

1500	C	8	30	5.9	4.7	0.37	0.62	8.3	0.96

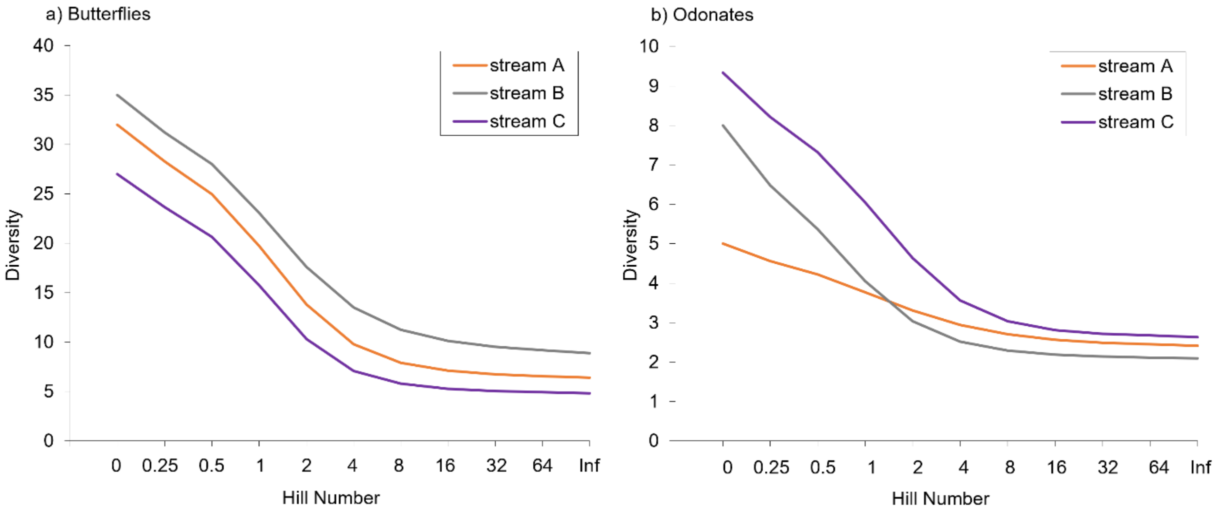

Figure S1. Alpha diversity per stream for butterflies (a) and odonates (b) represented by Hill numbers. Stream name codes mean: A-Fervença, B-Caniça and C-Loriga.
